# Editorial: Potential therapeutic targets in alcohol use disorder: insights from recent preclinical studies

**DOI:** 10.3389/fphar.2026.2015630

**Published:** 2026-10-19

**Authors:** Ilaria Morella, Priscila Vázquez-León, Valentina Vengeliene, Abraham Miranda-Páez

**Affiliations:** 1 Università di Pavia, Dipartimento di Biologia e Biotecnologie “Lazzaro Spallanzani”, Pavia, Italy; 2 Colegio de Ciencias y Humanidades, Academia de Biología Humana, Universidad Autónoma de la Ciudad de México, Plantel Cuautepec, Mexico City, Mexico; 3 Department of Neurobiology and Biophysics, Institute of Biosciences, Life Sciences Center, Vilnius University, Vilnius, Lithuania; 4 Departamento de Fisiología, Escuela Nacional de Ciencias Biológicas, Instituto Politécnico Nacional, Vilnius, Mexico

**Keywords:** alcohol use disorder, preclinical and clinical studies, neurotransmitters, therapeutic targets, alcohol self-administration

This Research Topic is a collection of articles addressing emerging pharmacological targets involved in Alcohol Use Disorder (AUD), with an emphasis on neurotransmitter systems that are implicated in alcohol reward, craving and relapse. AUD can be conceptualized as a progressive cycle of dysregulation in brain reward and anti-reward systems. Repeated alcohol use increasingly disrupts these systems, leading to reduced reward sensitivity, negative emotional states, compulsive alcohol use, and loss of control over drinking despite adverse consequences (Koob and Le Moal, 2001).

AUD represents a major public health concern since it can lead to substantial impairment in daily functioning while exacerbating the risk of chronic diseases, including stroke, cirrhosis, heart disease and mental health issues. Furthermore, AUD increases the likelihood of accidents, injuries and violence (GBD, 2016 Alcohol Collaborators, 2018). Although contradictory data might be found in recent published literature, ethanol consumption seems alarmingly widespread in non-traditional demographics such as underage and women (White et al., 2015; Esposito et al., 2025).

The neurobiological mechanisms underlying AUD are multifaceted as they involve multiple neurotransmitter systems. This Research Topic mainly focused on monoaminergic systems such as dopamine, serotonin, and glycine, and neuropeptidergic as opioids, ghrelin, neuropeptide-Y, and orexins. However, other neurotransmitter systems have been explored where emerging evidence indicated their potential involvement in AUD. It is worth noting that the complex interactions between genetic, neurobiological and environmental factors contributing to the development and persistence of AUD complicate the identification of novel therapeutics (Zhou and Gelernter, 2024; Lovinger and Roberto, 2025).

In this Research Topic, we present a study by Cui et al. assessing trends in the burden of AUD among people aged 15–64 years from 1990 to 2021 at the global, regional, and national levels, as well as projecting future trends. The authors conclude that AUD remains a significant contributor to the global disease burden, not only in the past but also in the years ahead, with serious implications for the working population, their families, and the broader socio-economic development. Despite the decline in AUD in the past 30 years, notable regional disparities, as well as differences across genders and ages, still persist, underscoring the need of targeted preventive strategies and treatment interventions.

Acute alcohol withdrawal (AAW) is a serious condition associated with increased incidence of other health conditions, including pneumonia and delirium tremens, leading to a heavy burden on healthcare services. Chen et al. Studied the relationship between sarcopenia, pneumonia and delirium, in 553 patients with excessive alcohol consumption at a psychiatric hospital in western China, from 2014 to 2023. The authors found that the blood-based indices associated with sarcopenia were linked to the risk of pneumonia and delirium among people hospitalized for AAW. These findings highlight the potential value of blood-based indices of sarcopenia to identify patients at risk of complications during AAW.

Despite the global burden of AUD, treatment options remain limited, highlighting the need for efficient and affordable therapeutic strategies. The rewarding and reinforcing effects of alcohol have been mainly attributed to the activation of the mesolimbic dopaminergic system. Although activation of accumbal glycine receptors (GlyRs) is required for alcohol-induced dopamine signaling, accumulating evidence indicates that additional neurotransmitter systems may be involved in this process. In this context, Cadeddu et al. demonstrate that GABA-rho receptors in the nucleus accumbens (NAc) contribute to the alcohol-induced increase in both dopamine and the endogenous GlyR agonists glycine and taurine. These findings identify GABA-rho receptors as a potential pharmacological target to treat AUD.

Recently, both monoaminergic and peptidergic neurotransmitter systems have attracted increasing attention for the study and treatment of AUD. Here, we present a recent study by Cantero-García et al. demonstrating that a single intranasal infusion of the neuropeptide galanin (1–15) in male adult Sprague-Dawley rats efficiently reduces alcohol self-administration and depressive-like behavior, with a favorable safety profile.

Studies in rodents previously identified dopamine and corticotropin-releasing hormone (CRH) systems, and amygdala circuits as important contributors to drug and alcohol reward, craving, and relapse. By using a combination of *in vivo* pharmacology, genetic targeting and behavioral studies, Broccoli et al. demonstrated an interaction between dopamine receptor-1 (D1) and corticotropin releasing hormone receptor-1 (CRHR1) in the intercalating cell masses in the amygdala. This interaction is critically involved in maladaptive stress coping, alcohol behavior and relapse, suggesting a novel mechanism potentially amenable to pharmacological intervention.

Finally, we present two studies addressing the effects of fenofibrate, a peroxisome proliferator-activated receptor alpha (PPAR-α) agonist already used for the treatment of dyslipidemia. In a brief research report, León et al. found that fenofibrate treatment during withdrawal reverses symptoms of ethanol-induced depression in male Sprague-Dawley rats. In addition, another work by Isla et al. demonstrates that fenofibrate can restore the homeostasis of monoaminergic, glutamate and dopamine systems in the NAc, and significantly reduce alcohol relapse in a model using rats selectively bred for high alcohol consumption. Altogether, these findings highlight PPAR-α activation as a potential therapeutic target for AUD.

Taken together, the studies presented in this Research Topic highlight the complex nature of AUD and the need to find novel pharmacological targets beyond the classical neurotransmitter systems.
